# Special Issue “Amino Acids and Related Compounds in Health and Disease”

**DOI:** 10.3390/ijms27010036

**Published:** 2025-12-19

**Authors:** Natalia Kurhaluk

**Affiliations:** Institute of Biology, Pomeranian University in Słupsk, Arciszewski Str. 22b, 76-200 Slupsk, Poland; natalia.kurhaluk@upsl.edu.pl

The following overview seeks to integrate and critically examine the diverse and intricately detailed findings presented across the contributions to the Special Issue “Amino Acids and Related Compounds in Health and Disease” in IJMS. The aim is to highlight the complex and, in many aspects, still incompletely understood roles of amino acids and their related compounds in the physiological and pathological processes that govern health and disease across a wide range of biological systems, including humans, animals, and plants. It is becoming increasingly evident that the established theoretical frameworks concerning amino acid metabolism, signalling, and homeostatic regulation are undergoing a substantial reconceptualisation. The extant evidence suggests that the dualistic nature of amino acid metabolism, in which protective and deleterious outcomes may coexist contingent upon the intricate balance of enzymatic flux, cofactor availability, cellular compartmentalisation, and physiological context, thereby underscores the necessity for a more nuanced, integrative, and dynamically responsive framework in future investigations aiming to fully decipher the complexity of amino acid-driven biological processes. This is being driven by the convergence of novel biochemical methodologies, advanced molecular profiling techniques, and integrative systems biology approaches. These have enabled researchers to uncover subtle yet significant layers of metabolic cross-talk, redox-dependent modulation, and context-specific adaptive responses that redefine the biological significance of these fundamental molecular entities.

Within this framework, the study conducted by Yuan et al. [1], representing the first contribution to the Special Issue, establishes a fundamental reference point for future research on the role of taurine in animal health and aquaculture innovation and is particularly significant for advancing our understanding of the multifaceted role of taurine in regulating physiological functions, especially in aquatic animals exposed to environmental stressors such as hypoxia and intensive farming practices. The researchers comprehensively investigated the effects of taurine supplementation on the gut microbiota composition and functionality in the critically endangered Chinese stripe-necked turtle (*Mauremys sinensis*), demonstrating that taurine can enhance immune responses and physiological resilience, while simultaneously exerting a modulatory influence on the diversity and structure of intestinal microbial communities. By integrating insights from immunology, microbiology, and aquaculture practices, this study provides a novel and scientifically grounded rationale for the use of taurine as a functional feed additive, offering practical implications for sustainable turtle farming and species conservation strategies.

The study by Casas-Barragán et al. [2], which is featured as the second article in this Special Issue, provides new insights into the molecular interplay between circulating amino acids and thermoregulation in fibromyalgia syndrome (FMS). Their research discloses discernible correlation patterns between amino acids, including tryptophan, methionine, and leucine, and both peripheral and core body temperatures. These patterns diverge significantly from those observed in healthy individuals. This finding suggests that amino acid metabolism may be fundamentally altered in FMS, potentially contributing to the thermoregulatory dysfunction that is characteristic of the condition. On a molecular level, these amino acids have been demonstrated to play a regulatory role in vascular tone and brown adipose tissue metabolism, both of which are critical for maintaining body temperature. The authors propose that immune-related fragmentation of transfer RNAs in FMS patients could reduce the availability of certain amino acids, thereby disrupting protein synthesis and cellular function that serve to deepen our understanding of the biochemical mechanisms underlying FMS symptoms, with a particular focus on impaired thermal sensation.

The third paper published in this Special Issue deserves particular attention as it provides an in-depth analysis of the interactions between the catecholamine system, its biosynthetic precursors, and the parasympathetic nervous system, all linked through the nitric oxide mechanism in the conditions of emotional stress. The authors investigate how modulation of these systems affects oxygen-dependent processes and the organism’s stress response, offering valuable insights into the complex regulation of stress at molecular and systemic levels. Combining biochemical analysis with mitochondrial function studies significantly advances our understanding of the protective and adaptive mechanisms that may have practical therapeutic implications for stress management [3]. It is important to note that the parasympathetic nervous system, specifically via the vagus nerve, functions as a primary counter-regulatory mechanism to the HPA axis. While sympathetic activation drives the “fight-or-flight” response, the parasympathetic system facilitates “rest-and-digest” processes, including metabolic recovery, anti-inflammatory signalling, and emotional regulation [4]. Vagal tone, which is a reflection of parasympathetic activity, has been shown to be inversely correlated with cortisol levels and psychological stress markers. Namely, a higher vagal tone has been demonstrated to be associated with greater emotional resilience and faster recovery from stress, especially in conditions that emphasise the role of gastrointestinal vagus nerve signalling in regulating neurocognitive processes that influence a range of adaptive behavioural responses [5,6].

As the primary neurotransmitter of the parasympathetic nervous system, acetylcholine (ACh) also affects cellular metabolism by stimulating the functionalities of mitochondrial enzymes and enhancing bioenergetic processes. It has been shown that there is a dynamic interaction between energy metabolism functionality, hormonal signalling, and cellular receptor systems, particularly in hypoxic conditions [7]. This is consistent with the hypothesis of a functional feedback loop wherein succinate (SC) oxidation is regulated by catecholamines, while exogenous succinate, in turn, stimulates catecholamine metabolism [8,9].

This bidirectional relationship suggests a direct role of succinate in regulating synaptic transmission. Additionally, ACh can activate the oxidation of α-ketoglutarate in mitochondria via stimulation of aminotransferase reactions, while simultaneously inhibiting succinate dehydrogenase (SDH) activity. This mechanism supports the concept of a “fast cycle” within the tricarboxylic acid cycle, which is activated under various types of functional load (hypoxia, stress, adaptation, diseases of different origin), and aligns with findings on the cholinomimetic properties of α-ketoglutarate [5,9]. These dependencies are shown in Figure 1.

This Special Issue focuses on the effects of various amino acids that have been analysed, highlighting their critical role in modulating metabolic processes and shaping physiological responses that help the body adapt, with particular emphasis on their influence on gut microbiome homeostasis. It is important to note that these amino acids interact with intestinal microbiota, supporting microbial ecosystem balance and enhancing parasympathetic nervous system activity via neuroimmune and neuroendocrine mechanisms. This interaction improves immune function, reduces inflammation, and optimises adaptive responses to environmental and physiological stressors.

The way in which ACh and nitric oxide (NO) interact, as demonstrated earlier in human skin tissue by the same authors [10], is crucial to the regulation of microcirculation. Here, ACh, released by the sympathetic cholinergic system, causes the blood vessels to widen, while NO plays a key role as a mediator that is produced by the endothelium and is involved in the control of blood flow and vasomotion. Another research has shown that ACh activates oxidative pathways that are not directly coupled with oxidative phosphorylation. Moreover, in hypoxic conditions, the shift in cellular respiration toward a nitrate–nitrite-dependent component increases survival rates in animals experiencing acute hypoxia [3].

The functional state of cholinergic receptors is therefore crucial for both cellular and mitochondrial metabolic remodelling in response to oxygen deficiency, not only in the context of ACh but also NO, the concentration of which significantly increases during adaptation to hypoxia [11]. Namely, high-intensity exercise performed in hypoxic conditions significantly enhances endothelial function by increasing NO bioavailability, as demonstrated in C57BL/6 mice where hypoxic exercise improved acetylcholine-induced vasorelaxation and reduced oxidative stress markers compared to normoxic exercise, independently of intensity, with the greatest effects observed at maximal and supramaximal intensities [11]. These results suggest that hypoxic high-intensity training could modulate the activity of endothelial NOS and antioxidant defences, thereby improving vascular reactivity and providing a promising approach to preventing cardiovascular disease.

The experimental results indicated that hypoxia significantly disrupts endothelial NO bioavailability by impairing key metabolic pathways, including the depletion of the essential eNOS cofactor tetrahydrobiopterin (BH4) and substrate L-arginine, which leads to eNOS uncoupling and a shift from NO to superoxide production. This metabolic dysfunction enhances oxidative stress and inflammation, ultimately driving endothelial dysfunction and contributing to the development of cardiovascular disease [12]. Thus, altered endothelial metabolism in hypoxic conditions is a central mechanism linking oxygen deficiency to reduced NO signalling and vascular pathology.

The study demonstrated that the fundamental physiological effect of ACh is the optimisation of oxygen uptake and utilisation under extreme hypoxic stress, and parasympathetic regulation appears to shape individual resistance to hypoxia, as confirmed by earlier studies [3,13]. From this perspective, we propose a general model for mitochondrial and cellular function regulation mediated not only by macroergic compounds but also by cofactors, metabolites, hormones, and second messengers. The reciprocity of these regulatory systems is essential for maintaining oxygen consumption and maximising its efficiency in ATP synthesis, in accordance with the cell’s functional state [14,15]. Further studies confirmed the role of ACh as an endogenous vasoactive agent that stimulates NO production, thereby outlining its role in adaptive strategies to hypoxia and other extreme conditions.

The available data lend support to the conclusion that the physiological individuality in response to hypoxic stress is also reflected in mitochondrial respiration. Namely, animals with high and low resistance to oxygen deficiency exhibit distinct profiles of alanine/aspartate aminotransferase and succinate dehydrogenase pathway activity, along with variable coupling efficiency between respiration and oxidative phosphorylation in cardiac and hepatic tissues, as described earlier in this article. Despite a decrease in the ADP/O ratio—an indicator of mitochondrial respiratory efficiency—high-resistance animals preserved high levels of respiration–phosphorylation coupling, suggesting a more efficient use of oxygen. Therefore, given the growing evidence on the interplay between parasympathetic regulation and the microbiome [16], it is plausible that the predisposition to activate these metabolic pathways represents a central mechanism of stress resistance. Therefore, the present findings, supported by the existing literature, underline the central role of ACh as a regulator of cellular and mitochondrial metabolism [17], particularly in conditions of oxidative stress and hypoxia. ACh modulates intracellular signalling pathways involving calcium, cyclic nucleotides, and oxidative phosphorylation, thereby influencing not only localised bioenergetic processes but also the systemic adaptive response [18,19]. The observed interactions between neurotransmission, mitochondrial enzymatic activity, and hormonal signalling highlight a complex reciprocal regulatory network that ensures metabolic flexibility in response to environmental challenges, introducing the biological role of non-neuronal acetylcholine in plants, humans, and gut microbiota [20,21,22].

The fourth study provides a comprehensive overview of the latest research findings in this Special Issue, offering significant mechanistic insights into the influence of dietary amino acids on liver metabolism. The study demonstrates that threonine deficiency in primary duck hepatocytes leads to excessive triglyceride accumulation by suppressing STAT3 phosphorylation, which in turn promotes lipid synthesis and inhibits lipid breakdown [23]. The authors’ findings are of particular importance because they identify STAT3 as a key mediator of the regulatory effects of threonine on hepatic lipid homeostasis, offering valuable implications for nutritional strategies in poultry production and potentially informing research on amino acid-related metabolic disorders in other species [23].

In light of the comprehensive findings compiled within this Special Issue, it is becoming increasingly evident that the pathophysiological implications of amino acid dysregulation—whether manifesting through aberrant catabolic fluxes, defective transporter functions or maladaptive signalling cascades—resonate across a wide spectrum of disease phenotypes. These range from neurodegenerative disorders, where excitotoxicity, neurotransmitter imbalances and oxidative stress are associated with metabolic insufficiencies, to systemic metabolic disorders such as diabetes mellitus, cardiovascular diseases and cancer, in which amino acid-derived metabolites act as active mediators and amplifiers of disease progression as well as passive biomarkers, thereby magnifying the need to use these biochemical nodes as leverage points for diagnostic innovation, prognostic accuracy and therapeutic precision.

Therefore, among the contributions to this Special Issue, the two review articles have generated the greatest interest, reflecting their wide-ranging relevance and practical significance in both clinical and nutritional research. Namely, the systematic review by Flores-Hernández et al. [24] convincingly demonstrates that high-protein diets can effectively reduce hyperglycaemia in patients with type 2 diabetes mellitus, emphasising the role of branched-chain amino acids, particularly leucine, in enhancing glucose metabolism and improving hepatic insulin sensitivity, thus providing evidence for a dietary strategy with direct clinical implications. In parallel, the review by Holeček [25] offers a detailed mechanistic examination of alanine and glutamine as key glucogenic amino acids, elucidating their complementary contributions to gluconeogenesis in the liver, kidneys, and small intestine in both physiological and pathological conditions, and highlighting their importance for energy homeostasis and metabolic adaptation during such states as fasting, high-protein feeding, and metabolic disorders. Collectively, these reviews illustrate the translational significance of amino acid metabolism, bridging fundamental biochemical insights with potential therapeutic and dietary applications, which explains the exceptional attention they have received from the scientific community [24,25].

A thorough examination of the interesting data presented in this IJMS Special Issue reveals a wealth of unique insights into a range of topics, including the antioxidative and non-antioxidative defence mechanisms mediated by amino acid derivatives, the intricate interplay between amino acid biosynthetic pathways and environmental stressors, and the translational relevance of amino acid-targeted interventions in the therapeutic landscapes of oncology, neurology, immunology, and metabolic syndromes. Hence, a unifying theme distinctly emerges, namely, that amino acids transcend their traditional designation as mere metabolic intermediates or structural constituents of proteins, asserting themselves instead as pivotal modulators of cellular homeostasis, intercellular communication, and organismal resilience in the face of both endogenous metabolic imbalances and exogenous environmental challenges.

It is evident that the collaborative efforts of the contributors to this IJMS Special Issue represent a substantial advancement in the existing body of research on amino acids. Instead, they delineate evolving conceptual landscape studies, wherein the integrative analysis of amino acid-related processes is increasingly recognised as indispensable to decoding the intricate, and often bidirectional, relationships between molecular perturbations and clinical manifestations, thereby charting a course for future research endeavours aimed at exploiting these critical molecular interfaces for the development of targeted interventions, precision medicine strategies, and holistic therapeutic frameworks that transcend conventional disciplinary boundaries.

The success of this Special Issue is attributable to the collective dedication and intellectual enquiry of the researchers who have successfully navigated the complex interplay of the experimental design, data interpretation, and theoretical reflection, allowing this Special Issue to present a coherent body of work, offering novel insights into amino acid metabolism, signalling pathways, oxidative stress modulation, and clinical applications, thereby setting a benchmark for future investigations in the field. The Guest Editor of this Special Issue wishes to extend their sincere appreciation to all authors whose valuable contributions and high-quality research have significantly enriched this publication and would also like to express their gratitude to the reviewers for their meticulous and constructive feedback, which guaranteed the scientific rigour and quality of the articles.

## Figures and Tables

**Figure 1:**
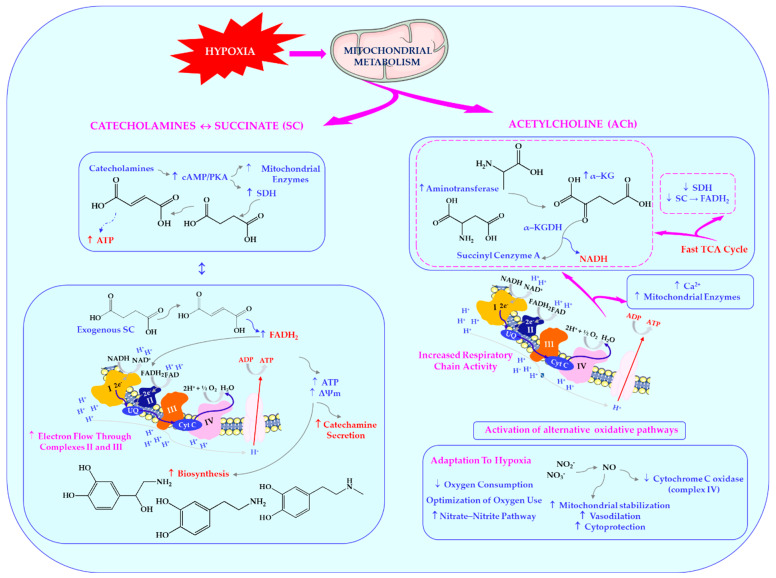
Succinate and α-ketoglutarate in the receptor-mediated metabolic control of mitochondrial function and the Krebs cycle during stress. In stress conditions, a complex and bidirectional interaction emerges between cellular energy metabolism, hormonal signalling pathways, and receptor-mediated processes, reflecting the integrated response of the organism to oxygen deficiency and the necessity for rapid adaptive mechanisms. L-arginine, functioning as the principal substrate for nitric oxide (NO) synthesis, plays a pivotal role in facilitating mitochondrial adaptation, modulating vascular tone and blood flow, and regulating synaptic signalling, thereby contributing to the organism’s capacity to withstand hypoxic challenge. The oxidation of succinate (SC) is tightly regulated by catecholaminergic activity, while the administration of exogenous succinate has been shown to enhance catecholamine turnover, indicating a regulatory role for succinate in maintaining synaptic homeostasis in stress conditions. Acetylcholine (ACh) exerts a dual modulatory effect by stimulating α-ketoglutarate oxidation through aminotransferase activation and concurrently inhibiting succinate dehydrogenase, which together allow a rapid flux through the tricarboxylic acid cycle independent of oxidative phosphorylation. The functional integrity of ACh receptors and the capacity for NO generation emerge as critical determinants of individual resistance to hypoxia, highlighting the interplay between metabolic flexibility, neurotransmitter signalling, and vascular adaptation in shaping adaptive responses to oxygen deprivation. Abbreviations: Ach-acetylcholine, ADP-adenosine diphosphate, ATP-adenosine triphosphate; cAMP-cyclic adenosine monophosphate, Cyt C-cytochrome C, α-KG-α-ketoglutarate, α-KGDH-α-ketoglutarate dehydrogenase, FADH_2_-flavin adenine dinucleotide, NADH-nicotinamide adenine dinucleotide, NO-nitric oxide, PKA-protein kinase A, SC-succinate, SDH-succinate dehydrogenase, TCA-tricarboxylic acid cycle, UQ-ubiquinone, ΔΨm-mitochondrial membrane potential. This Figure was created using Servier Medical Art (available at https://smart.servier.com/) (accessed on 1 May 2025).
